# Rate dynamics of the retina-LGN connection

**DOI:** 10.1186/1471-2202-12-S1-P90

**Published:** 2011-07-18

**Authors:** Thomas Heiberg, Tom Tetzlaff, Birgit Kriener, Hans E Plesser, Gaute T Einevoll

**Affiliations:** 1 Dept. of Mathematical Sciences & Technology, Norwegian Univ. Life Sciences, 1432 Aas, Norway

## 

Firing-rate models provide a practical tool for studying the dynamics of trial- or population-averaged neuronal signals. The derivation or extraction of such models through investigation of the firing-rate response characteristics of ensembles of neurons has been the subject of several studies (see references in [1]). The majority of these focused on neurons that receive input spikes at a high rate through weak synapses (diffusion approximation). For many neural systems, however, this assumption cannot be justified. One example in the early visual system is the lateral geniculate nucleus (LGN), where synapses between retinal ganglion cells and relay cells are so strong that single retinal spikes can initiate action potentials in the thalamic targets.

**Figure 1:**
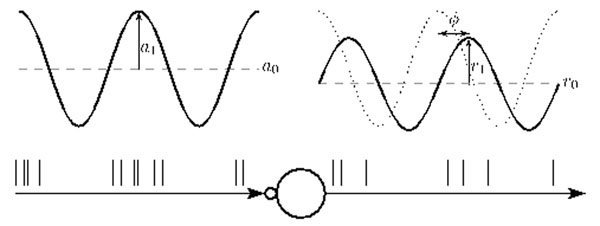
A neuron model (denoted by large circle) receives, through a synapse (small circle), spike trains generated by a Poisson point process with sinusoidal rate of mean a_0_ and modulation amplitude a_1_. The response firing rate is characterized by its mean r_0_, amplitude r_1_ and phase ϕ. Adapted from [1].

Using a comprehensive numerical approach, we recently studied the firing-rate response properties of leaky integrate-and-fire (LIF) neurons receiving current input through strong synapses [1]. Input spike trains were modeled as inhomogeneous Poisson point processes with sinusoidally modulated rate. Average rates, modulation amplitudes, and phases of the period-averaged spike responses were measured for a broad range of stimulus, synapse, and neuron parameters, cf Fig. 1. We found that the resulting responses could be described well by a linear first-order low-pass filter over a wide range of model parameters. Combining this filter with the nonlinear response characteristic for stationary inputs, we constructed a linear-nonlinear firing-rate model, which accurately predicted the population response for a variety of non-sinusoidal test stimuli.

In the present study, we use the same approach to investigate whether linear-nonlinear firing-rate models can capture equally well the firing rate properties of LGN relay neuron models that have been fitted to experimental data [2,3]. Models investigated include more “realistic” ones with conductance-based synaptic and after-hyperpolarizing currents [2] as well as more abstract spike-response models [3,4].
